# Reciprocity between a retrograde signal and a putative metalloprotease reconfigures plastidial metabolic and structural states

**DOI:** 10.1126/sciadv.abo0724

**Published:** 2022-06-03

**Authors:** Jin-Zheng Wang, Wilhelmina van de Ven, Yanmei Xiao, Xiang He, Haiyan Ke, Panyu Yang, Katayoon Dehesh

**Affiliations:** 1 Institute for Integrative Genome Biology and Department of Botany and Plant Sciences, University of California, Riverside, Riverside, CA 92521, USA.; 2 Department of Plant Biology, University of California, Davis, Davis, CA 95616, USA.

## Abstract

Reconfiguration of the plastidial proteome in response to environmental cues is central to tailoring adaptive responses. To define the underlying mechanisms and consequences of these reconfigurations, we performed a suppressor screen, using a mutant (*ceh1*) accumulating high levels of a plastidial retrograde signaling metabolite, MEcPP. We isolated a revertant partially suppressing the dwarf stature and high salicylic acid of *ceh1* and identified the mutation in a putative plastidial metalloprotease (VIR3). Biochemical analyses showed increased VIR3 levels in *ceh1*, accompanied by reduced abundance of VIR3-target enzymes, ascorbate peroxidase, and glyceraldehyde 3-phophate dehydrogenase B. These proteomic shifts elicited increased H_2_O_2_, salicylic acid, and MEcPP levels, as well as stromule formation. High light recapitulated VIR3-associated reconfiguration of plastidial metabolic and structural states. These results establish a link between a plastidial stress-inducible retrograde signaling metabolite and a putative metalloprotease and reveal how the reciprocity between the two components modulates plastidial metabolic and structural states, shaping adaptive responses.

## Teaser

Reciprocity between a stress-signaling metabolite and a protease modulates plastidial metabolic and structural states.

## INTRODUCTION

Plastids are metabolic, signaling, and sensing centers well equipped with a range of biochemical networks bound to maintain the quality and quantity of the plastidial proteome through regulated proteolysis and protein quality control mechanisms. To date, combined biochemical, genetic, and proteomic studies have identified about 20 chloroplast protein-degrading machineries with more than 50 constituents (*1*). Among them, a group of membrane-bound proteases known as FtsH (filamentation temperature sensitive) initially was identified in an *Escherichia coli* mutant with aberrant growth behavior (*2*). In plants, FtsHs are strictly present in the endosymbiotic-derived organelles, mitochondria, and chloroplasts. While *E. coli* contains only one FtsH, the *Arabidopsis* genome contains 17 *FtsH* sequences, 12 of which are known to code active enzymes and 8 of which are exclusively targeted to chloroplasts (*3*, *4*). Most of these proteases contain an AAA (adenosine triphosphatase associated with various cellular activities) domain and a metalloprotease domain ligating a Zn^2+^ ion in the consensus sequence HEXXH (where X is any uncharged residue) (*5*). In general, thylakoid localized FtsHs are critical in biogenesis of thylakoid membranes and are also implicated in retrograde signaling. One such example is FtsH2 (VAR2), proposed to be involved in retrograde signaling via EXECUTER1 (EX1)–dependent mechanisms, whereby degradation of EX1 by FtsH2 is necessary for activation of the retrograde signaling pathway (*6*, *7*).

Another member of metalloprotease superfamily is the stroma lamella localized VIRESCENT3 (VIR3) that lacks the adenosine 5′-triphosphate (ATP)–binding domain but has the signature zinc-binding motif HEXXH at the C terminus (^235^HEAGH^239^) (*8*–*10*). The zinc-binding site is exposed to stroma, and its perturbation by His^235^ substitution interferes with VIR3 function and/or stability (*10*). Loss of VIR3 function causes a virescent phenotype, hence the designation. It is suggested that VIR3 potentially exerts proteolytic activity as a monomer or in small complexes to maintain quality and quantity of the plastidial proteome (*10*).

Environmental perturbations result in increased production of reactive oxygen species (ROS) to lethal levels if unchecked. Ascorbate peroxidase (APX) is a multifamily of antioxidant isoenzymes originating from alternative splicing (*11*). The isoenzymes catalyze the conversion of H_2_O_2_ into H_2_O, under both standard and stress conditions in various subcellular compartments including cytosol, peroxisome, mitochondria, and chloroplast (stroma and membrane-bound thylakoid) (*12*–*14*). The thylakoidal isoform (tAPX) borders with the acceptor of photosystem I and as such the first enzyme to intercept an H_2_O_2_ molecule produced (*15*).

Glucose metabolism is the foundation of all biochemical activities, and glyceraldehyde 3-phosphate dehydrogenase (GAPDH) is the ubiquitous multifamily enzyme composed of glycolytic GAPDHs (GAPC) and highly regulated plastidial enzymes (*GAPA* and *GAPB*) catalyzing the conversion of 1, 3-bisphosphoglycerate (1,3BPG) to glyceraldehyde 3-phosphate (GAP) with concomitant reduction of NADPH (reduced nicotinamide adenine dinucleotide phosphate) to NADP^+^ in the photosynthetic reductive carbon cycle (*16*–*20*). In addition to their housekeeping roles, the GAPDHs are involved in nontraditional and diverse cellular functions such as influencing cell fate, regulation of ROS accumulation, and cell death in response to pathogen attack (*21*, *22*). One may further surmise expanded regulatory function of plastidial GAPDHs given that the first step of isoprenoids production by plastidial methylerythritol phosphate (MEP) pathway is the condensation of pyruvate and GAP (*23*–*26*).

Intriguingly, the MEP pathway not only serves as a central plastidial biochemical route but also functions as a sensing and signaling pathway confirmed by the functionality of its intermediate, 2-C-methyl-d-erythritol-2,4-cyclopyrophosphate (MEcPP), as a precursor of isoprenoids and as a stress-specific retrograde signaling metabolite coordinating expression of selected stress-response nuclear genes (*27*–*31*). The discovery of MEcPP as a retrograde signaling metabolite was through a genetic screen that led to the isolation of a mutant line designated *ceh1*, for constitutive expression of *hydroperoxide lyase* (*HPL*), an otherwise stress-inducible nuclear gene encoding a plastidial enzyme in the HPL branch of the oxylipin pathway (*30*). This mutation is caused by a single amino acid substitution in the penultimate MEP-pathway enzyme, hydroxymethylbutenyl diphosphate synthase (HDS), resulting in accumulation of MEcPP and the consequential growth retardation, in concert with high levels of salicylic acid (SA) and the induction of selected nuclear-encoded stress-response genes (*30*, *32*–*34*).

Toward identification of the genetic components of MEcPP signal transduction pathway, we performed a suppressor screen for revertants with fully or partially abolished *ceh1* phenotypes, despite high MEcPP levels. Here, we report reversion of selected *ceh1* mutant phenotypes by a mutant allele of *VIRESCENT3* (*VIR3*). Next, we establish the significance of zinc-binding motif in functional integrity of this putative metalloprotease and further identify VIR3 interacting partners. Through biochemical, metabolomics, and cell biological approaches, we found an inverse relationship between abundance of the enzyme and its interacting partners linked to a feedback loop for accumulation of MEcPP and, by extension, SA and accompanied by formation of stromules, proposed conduits for information exchange between chloroplast and nucleus. Last, high-light (HL) stress recapitulation of the VIR3-associated multicomponent modification illustrates the biological relevance of these findings. In summary, our results uncover a central component of the plastidial sensing, signaling, and biochemical activities, reshaping the metabolic and structural states of this organelle in response to adverse environmental inputs.

## RESULTS

### VIR3: A component of MEcPP signal transduction pathway

**Fig. 1.**
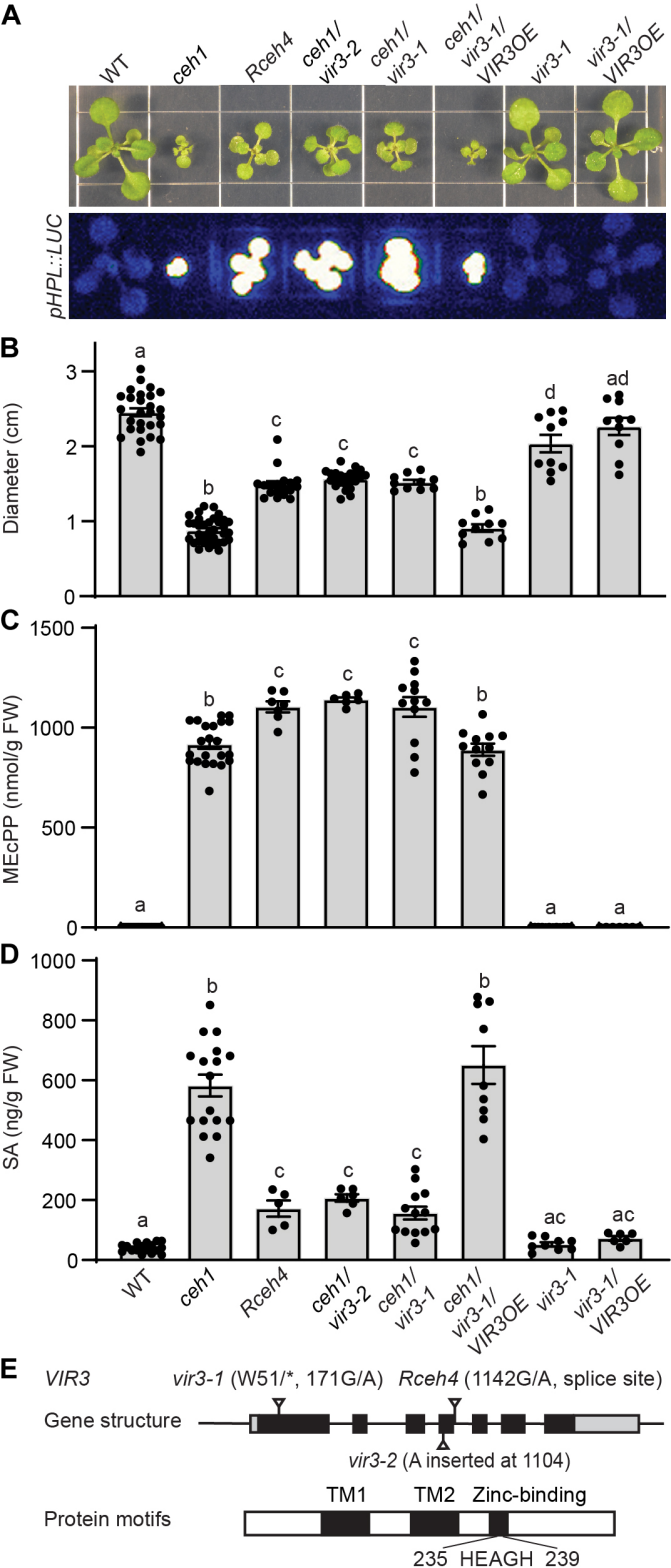
VIR3 is a component of MEcPP signal transduction pathway. (**A**) Representative images of 2-week-old wild-type (WT), *ceh1*, *Rceh4*, *ceh1/vir3-1*, *ceh1/vir3-2*, *ceh1/vir3-1/VIR3OE*, *vir3-1*, and *vir3-1/VIR3OE* seedlings grown in long days (16-hour light/8-hour dark) (top) and the corresponding dark-field images displaying *pHPL::LUC* activity (bottom). (**B**) Seedling diameter and (**C**) MEcPP and (**D**) SA levels of the aforementioned genotypes based on fresh weight (FW) of tissue. Data (B to D) are means ± SEM for each genotype with at least three biological replicates. Letters above bars indicate significant differences determined by one-way analysis of variance (ANOVA) with Tukey’s multiple comparisons test (*P* < 0.05). (**E**) Gene and protein structures of *VIR3*. Black boxes represent exons, lines represent introns and untranslated regions, and nucleotide and amino acid changes are displayed on the top or at the bottom of the top bar. The VIR3 transmembrane domains (TM) and the conserved zinc-binding domain (HEAGH) are shown on the bottom panel bar.

To identify the genetic components of the MEcPP-mediated signal transduction pathway, we mutagenized the *ceh1* mutant using ethyl methanesulfonate (EMS) and searched for revertants (*Rceh*) that, despite high MEcPP levels, display full or partial reversion of the *ceh1* phenotypes, such as reversion of dwarf stature, constitutive *HPL* expression, and high SA content. This led to identification of a mutant initially designated *Rceh4* (*revertant of ceh1 line 4*), displaying partial recovery of *ceh1* dwarfed stature by ~50% (Fig. 1, A and B). Targeted metabolomics analyses showed no reduction in the MEcPP levels but a notable reduction (~70%) in SA, albeit with no alteration in high and constitutive *HPL*-driven luciferase (LUC) activity, in the *Rceh4* compared to the *ceh1* mutant (Fig. 1, A, C, and D). Whole-genome sequencing identified *VIR3* as the candidate gene harboring single-point mutation at the fourth intron splicing site, thereby interfering with splicing of the intron and the consequential introduction of a premature stop codon into the encoded protein (Fig. 1E and fig. S1, A to C). Bioinformatics studies identified the encoded protein as a member of metalloprotease superfamily lacking the ATP-binding domain but containing the signature zinc-binding motif HEXXH at the C terminus (^235^HEAGH^239^) (Fig. 1E) (*8*–*10*).

To confirm *VIR3* as the gene responsible for partial reversion of *ceh1* aberrant phenotypes, we crossed *ceh1* to *vir3-1* loss of function mutant (*ceh1*/*vir3-1)* (Fig. 1E). In addition, we generated *ceh1* lines expressing clustered regularly interspaced short palindromic repeats (CRISPR)–based construct with guide RNA ~30 base pairs upstream of *Rceh4* point mutation (herein designated *ceh1/vir3-2*). The *ceh1*/*vir3-2* expresses a premature VIR3 protein as the result of a nucleotide insertion at the fourth exon (Fig. 1E and fig. S1, B to D). The phenotypic and metabolomics analyses of *ceh1* backgrounds introgressed into two independent *vir3* loss of function mutants (*ceh1/vir3-1* and *ceh1/vir3-2*) matched those of the *Rceh4*, thereby confirming *vir3* as the mutant allele responsible for suppressing the dwarf stature and SA level phenotypes of the *ceh1* mutant in the revertant, albeit with no impact on the constitutive expression of *HPL* (Fig. 1, A to E). We further verified the finding by integrating *vir3-1/p35::VIR3-FLAG* (*vir3-1/VIR3OE)* (a gift from F. Yu) into the *ceh1* mutant backgrounds (*ceh1/vir3-1/VIR3OE*). The data show that similarly to the *ceh1* mutant, *ceh1/vir3-1* seedlings overexpressing *VIR3* are dwarfed and contain high SA levels (Fig. 1, A to D). This further verifies the mutation in *VIR3* responsible for partial suppression of *ceh1* phenotypes in the *Rceh4* suppressor line. It is noteworthy that all the analyses indicate that under standard growth conditions, *vir3-1* and *vir3-1*/*VIR3OE* lines are indistinguishable from each other and from the wild-type (WT) plants, supporting the functional association of VIR3 with stress, as evidenced by VIR3 functional input in the high MEcPP–accumulating *ceh1* line.

**Fig. 2.**
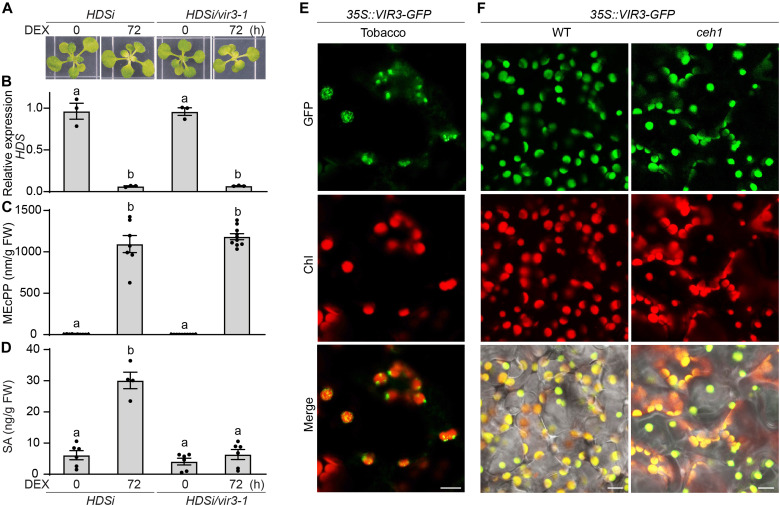
VIR3 is essential for MEcPP-mediated SA accumulation. (**A**) Representative images of 2-week-old untreated (0) or DEX-treated (after 72 hours) *HDSi* and *HDSi/vir3-1* seedlings grown under long-day conditions. (**B**) Relative expression levels of *HDS* in untreated (0) or DEX-treated (after 72 hours) genotypes. (**C** and **D**) Levels of MEcPP (C) and SA (D) in the aforementioned genotypes based on fresh weight (FW) of tissue. Data (B to D) are means ± SEM for each genotype with at least three biological replicates. Statistical analyses were performed with one-way ANOVA with Tukey’s multiple comparisons test (*P* < 0.05). (**E** and **F**) Plastidial localization of VIR3 in tobacco leaves transiently expressing *VIR3-GFP* (scale bar, 10 μm) (E) and in the stably transformed WT and *ceh1* seedlings (scale bars, 5 μm) (F), displaying the merger of the green fluorescent protein (GFP) signal with the plastidial fluorescence.

To assess the potential interference of constitutively high MEcPP levels with the signal transduction pathway components, we further analyzed the dexamethasone (DEX)-inducible MEcPP-accumulating genotypes in the WT (*HDSi*) and *vir3* mutant backgrounds (*HDSi/vir3-1*). At 72 hours after DEX induction, seedlings display equally chlorotic phenotypes, together with reduced expression of *HDS* and in concert with enhanced MEcPP levels in both genotypes (Fig. 2, A to C). In contrast, however, DEX-induced MEcPP-mediated increase in SA levels remained restricted to the *HDSi* line and was only maintained at the basal levels in the *HDSi/vir3-1* (Fig. 2D). These data confirm VIR3 as a component of MEcPP signal transduction pathway that can limit SA production.

To confirm plastidial localization of VIR3, we first imaged VIR3 localization in tobacco leaves transiently expressing 35S::VIR3-GFP, using confocal microscopy. The images show plastidial localization of VIR3 (Fig. 2E). Next, to exclude the potential mislocalization of VIR3 as the result of high MEcPP levels in the *ceh1* mutant background, we examined and confirmed plastidial localization of VIR3 in the WT as well as in the mutant expressing 35S::VIR3-GFP (Fig. 2F). Collectively, the data verify the previously reported plastidial localization of VIR3 (*10*) and further confirm its unperturbed localization by high MEcPP content in the *ceh1* mutant.

### VIR3 function depends on the integrity of the zinc-binding motif

**Fig. 3.**
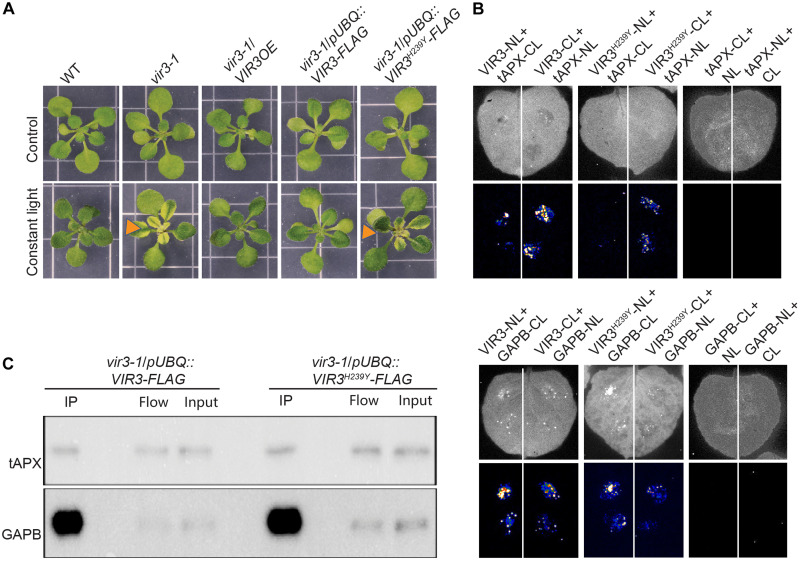
Zinc-binding motif is required for the functionality but not for VIR3 binding to tAPX and GAPB. (**A**) Representative images of WT, *vir3-1*, *vir3-1/VIR3OE*, *vir3-1/pUBQ::VIR3-FLAG*, and *vir3-1/pUBQ::VIR3^H239Y^-FLAG* seedlings grown in long days (16-hour light/8-hour dark), without (control) or with exposure to 7 days of constant light leading to the exclusive development of variegation patterns on *vir3-1* and *vir3-1/pUBQ::VIR3^H239Y^-FLAG* leaves but not in the other genotypes. (**B**) Representative images of infiltrated *N. benthamiana* leaves (top) and the corresponding split luciferase (LUC) complementation assays (bottom), expressing permutations of VIR3 or VIR3^H239Y^ with tAPX (top) or GAPB (bottom), and each fused to N- and C-terminal fragments of LUC (NL and CL, respectively). Controls consisted of LUC fusion constructs between tAPX and GAPB. LUC luminescence was analyzed after 2 days. The experiments were repeated at least three times. (**C**) Co-IP assay displays the in vivo interaction between VIR3 with tAPX and GAPB in *vir3-1/pUBQ::VIR3-FLAG* and *vir3-1/pUBQ::VIR3^H239T^-FLAG* seedlings, using FLAG antibody for Co-IP, and tAPX and GAPB- specific antibodies for immunoblot assays. The lanes between IP and flow are blank.

To test the functional significance of the C-terminal zinc-binding motif (^235^HEAGH^239^) of VIR3, we generated transgenic *vir3-1* lines expressing VIR3-FLAG and VIR3^H239Y^-FLAG constructs driven by ubiquitin 10 (UBQ10) promoter. We specifically exploited UBQ10-driven expression construct of VIR3, because the weak expression levels driven by the native promoter proved prohibitive to the studies. Next, we examined the phenotypic characteristics of these genotypes along with the corresponding controls, namely, WT, *vir-3*, and *vir3-1*/*VIR3OE*, under standard and constant light conditions. In contrast to the indistinguishable phenotypic characteristics of all the examined genotypes grown under standard conditions, the *vir3-1* and *vir3*-1/*pUBQ::VIR3^H239Y^-FLAG* genotypes displayed distinct variegation patterns not observed in the remainder lines grown under continuous light conditions (Fig. 3A). The data illustrate the significance of zinc-binding domain for VIR3 functionality and further denote the stress-associated function of the enzyme.

### VIR3 physically interacts with tAPX and GAPB

The significance of the zinc-binding domain in VIR3 functionality strengthened the notion of its putative function as a metalloprotease. It is of note that the nature of VIR3 as a membrane-bound protein impeded our efforts to examine the protease activity of recombinant VIR3 enzyme in an in vitro activity assay. However, the functionality of zinc-binding domain led us to question the existence of VIR3 interactive/substrate proteins. Toward this goal, we used *Nicotiana benthamiana* leaves transiently expressing *pUBQ::VIR3-FLAG* and *pUBQ::VIR3^H239Y^-FLAG* to immunoprecipitate VIR3 interacting proteins using the FLAG antibody, followed by liquid chromatography–mass spectrometry (LC-MS) analyses of the candidate proteins. Both constructs led to immunoprecipitation of several common proteins, among them the two that we initially focused on: the thylakoidal isoform of APX (tAPX; At1G77490) and plastidial GAPDH isoform B (GAPB; At1G42970) (data S1). This focus is based on the function of tAPX as an H_2_O_2_ scavenger, and the GAPB enzyme catalyzing the conversion of 1,3BPG to GAP, the MEP pathway–initiating substrate.

Next, we exploited split LUC complementation assays in tobacco leaves transiently expressing different combinations of native and mutated *VIR3*/*tAPX* and *VIR3*/*GAPB* constructs fused to N- or C-terminal fragment of *luciferase* (*LUC*). To eliminate possible autoluminescence, we checked LUC activity in leaves expressing *tAPX* and *GAPB* constructs fused to *LUC* (Fig. 3B). The bioluminescence data show reconstitution of LUC activity in leaves coinfiltrated with VIR3 or VIR3^H239Y^ together with tAPX or GAPB but not in the control leaves (Fig. 3B and fig. S2A). Specifically, both native VIR3 and VIR3^H239Y^ display notable physical interaction with tAPX and GAPB, with the strongest signals observed in leaves expressing VIR3-CL/tAPX-NL and in VIR3-NL/GAPB-CL expressing leaves.

To verify these interactions, we used *Arabidopsis vir3-1* lines expressing VIR3-FLAG and VIR3^H239Y^-FLAG and performed coimmunoprecipitation (Co-IP) assay using a FLAG antibody, followed by immunoblot analyses using the tAPX and GAPB-specific antibodies (Fig. 3C and fig. S2, B and C). The previously reported instability of VIR3 in transgenic lines expressing VIR3^H235L^-FLAG zinc-binding domain (*10*) led us to examine the integrity of the VIR3 and VIR3^H239Y^ proteins transiently expressed in *N. benthamiana* leaves, by immunoblot analyses using FLAG antibody (fig. S2B). The presence of VIR3 in both samples excludes the possibility of protein instability as the result of VIR3^H239Y^ mutation. Next, we performed immunoblot analyses using tAPX and GAPB-specific antibodies. The clear and specific presence of a tAPX and a GAPB reacting band in the IP fraction verified the in vivo interaction of VIR3 with the tAPX and GAPB proteins in stably transformed *Arabidopsis* lines (Fig. 3C and fig. S2C). As an additional control for excluding nonspecific binding, we performed the immunoblots using tAPX and GAPB-specific antibodies on beads incubated with protein extracts from nontransgenic WT seedlings (fig. S2D). Collectively, our data show that VIR3 physically interacts with tAPX and GAPB and further excludes the significance of the integrity of zinc-binding domain for the interaction while confirming significance of the zinc-binding domain for VIR3 functionality.

### MEcPP-mediated increase in VIR3 is inversely correlated with tAPX and GAPB abundance

**Fig. 4.**
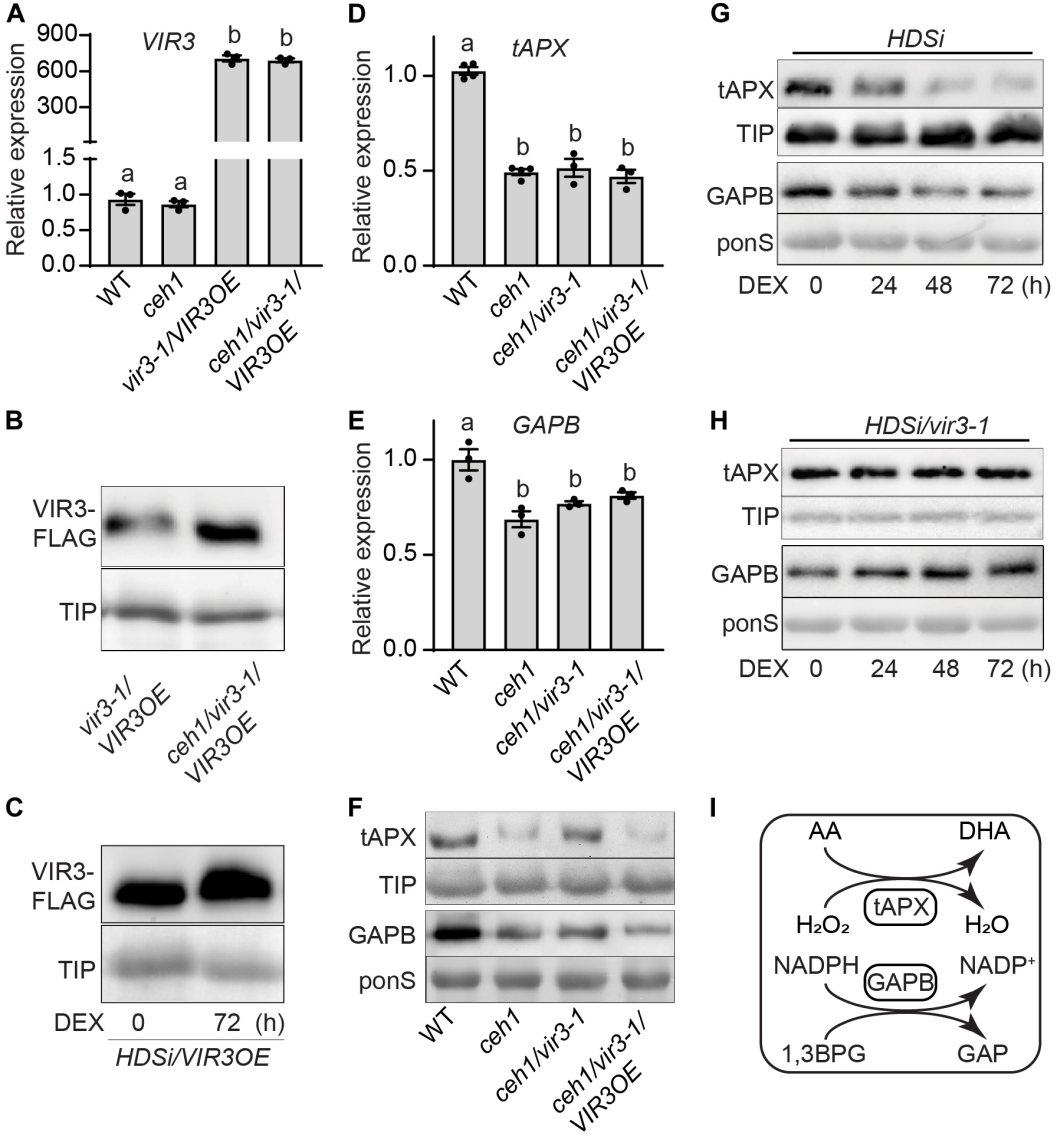
MEcPP-mediated increase in VIR3 is inversely correlated with tAPX and GAPB abundance. (**A**) Transcript levels of *VIR3* in WT, *ceh1*, *vir3-1/VIR3OE*, and *ceh1/vir3–1/VIR3OE* seedlings. (**B** and **C**) Immunoblot analyses of VIR3 abundance using FLAG antibody applied on blot of protein extracts from *vir3-1/VIR3OE* and *ceh1/vir3-1/VIR3OE* (B) and (C) from untreated (0) and DEX-treated (72 hours) *HDSi/VIR3OE* seedlings support MEcPP-mediated increase in VIR3 protein abundance. Specific antibody against TIP was used on the same blot to determine equal loading of the membrane-bound proteins. (**D** and **E**) Transcript levels of tAPX (D), and GAPB (E) in WT, *ceh1*, *vir3-1/VIR3OE*, and *ceh1/vir3-1/VIR3OE*. Total RNAs isolated from 2-week-old seedlings grown under long-day (16-hour light/8-hour dark) condition was subjected to real-time qRT-PCR (quantitative reverse-transcription polymerase chain reaction) analyses and subsequently normalized to the levels of At4g26410 (M3E9). Data are means ± SEM for each genotype with at least three biological replicates. Statistical analyses were performed with one-way ANOVA with Tukey’s multiple comparisons test; different letters indicate significant differences (*P* < 0.05). (**F**) Immunoblot analyses showing tAPX (top) and GAPB (bottom) protein abundance in WT, *ceh1*, *ceh1/vir3-1*, and *ceh1/vir3-1/VIR3OE*. Specific antibody against TIP was used on the tAPX blot to show equal loading of the membrane-bound protein, and Ponceau staining (ponS) was used for the loading control of the GAPB, as the soluble protein. (**G** and **H**) Immunoblot analyses displaying tAPX and GAPB abundance in *HDSi* (G) and *HDSi/vir3-1* genotypes (H), at various hours after DEX treatment (0, 24, 48, and 72 hours), using TIP and ponS as loading controls, respectively. (**I**) Schematic presentation of tAPX function in conversion of H_2_O_2_ to water along with conversion of ascorbic acid (AA) to dehydroascorbic acid (DHA); and GAPB catalyzing the conversion of 1,3BPG to GAP with concomitant reduction of NADPH to NADP^+^.

Partial and selective suppression of *ceh1* phenotypes in *Rceh4*, *ceh1*/*vir3-1*, and *ceh1*/*vir3-2* lines (Fig. 1, A to E) prompted the question of the potential impact of MEcPP accumulation on transcriptional and/or translational regulation of VIR3. To address this, we first compared VIR3 transcript levels in WT, *ceh1*, *vir3-1/VIR3OE*, and *ceh1/ vir3-1/VIR3OE* lines (Fig. 4A). The presence of similar *VIR3* transcript levels in *ceh1* versus the WT and between *vir3-1/VIR3OE* and *ceh1/ vir3-1/VIR3OE* lines is a clear indication of unaltered expression levels as the result of high MEcPP accumulation. However, immunoblot analyses of FLAG-tagged lines showed enhanced VIR3 abundance in *ceh1/vir3-1/VIR3OE* compared to the levels in *vir3-1/VIR3OE* lines (Fig. 4B and fig. S3, A and B). Additional immunoblots analyses using FLAG-tagged *HDSi*/*VIR3OE* lines before and 72 hours after DEX induction confirmed the positive correlation between increased MEcPP accumulation and enhanced VIR3 abundance (Fig. 4C and fig. S3, C and D).

Next, we examined transcriptional and translational profiles of tAPX and GAPB in WT, *ceh1*, *ceh1/ vir3-1*, and *ceh1/ vir3-1/VIR3OE* lines (Fig. 4, D to F, and fig. S3, E to G). This data shows reduced levels of *tAPX* and *GAPB* expression in all the constitutively MEcPP accumulating *ceh1* backgrounds compared to the WT (Fig. 4, D and E, and fig. S3, F and G). The reduced expression levels in all the *ceh1* backgrounds are independent of the presence or absence of VIR3. In contrast, the tAPX and GAPB protein levels appear to be independent of the *ceh1* mutant background but dependent on the presence of VIR3 (Fig. 4F and fig. S3E). The data specifically show a decrease in the abundance of both tAPX and GAPB proteins in *ceh1* and *ceh1*/*vir3-1*/*VIR3OE* compared to the WT and *ceh1*/*vir3-1*, thereby identifying VIR3 as a potential metalloprotease targeting these binding partners, albeit at different degrees.

Given the inverse relationship between the abundance of VIR3 and the levels of its binding proteins in the *ceh1* mutant background, we further examined the validity of this finding by testing tAPX and GAPB protein levels in the inducible MEcPP-producing lines, *HDSi* and *HDSi*/*vir3-1* (Fig. 4, G and H, and fig. S3, H to M). These data show reduced tAPX and GAPB protein levels after DEX induction in *HDSi* but not in *HDSi/vir3-1* lines, thereby providing additional support to the notion of VIR3 function as a metalloprotease responsible for the degradation of these binding partners/substrates. Collectively, the data provide experimental evidence for the likely MEcPP-mediated enhanced abundance/stability of VIR3 protein, but not the corresponding transcript levels in the *ceh1* mutant background, and further illustrate an inverse correlation between the abundance of VIR3 and its binding proteins, tAPX and GAPB.

### VIR3 functional input in ROS production and stromule formation

**Fig. 5.**
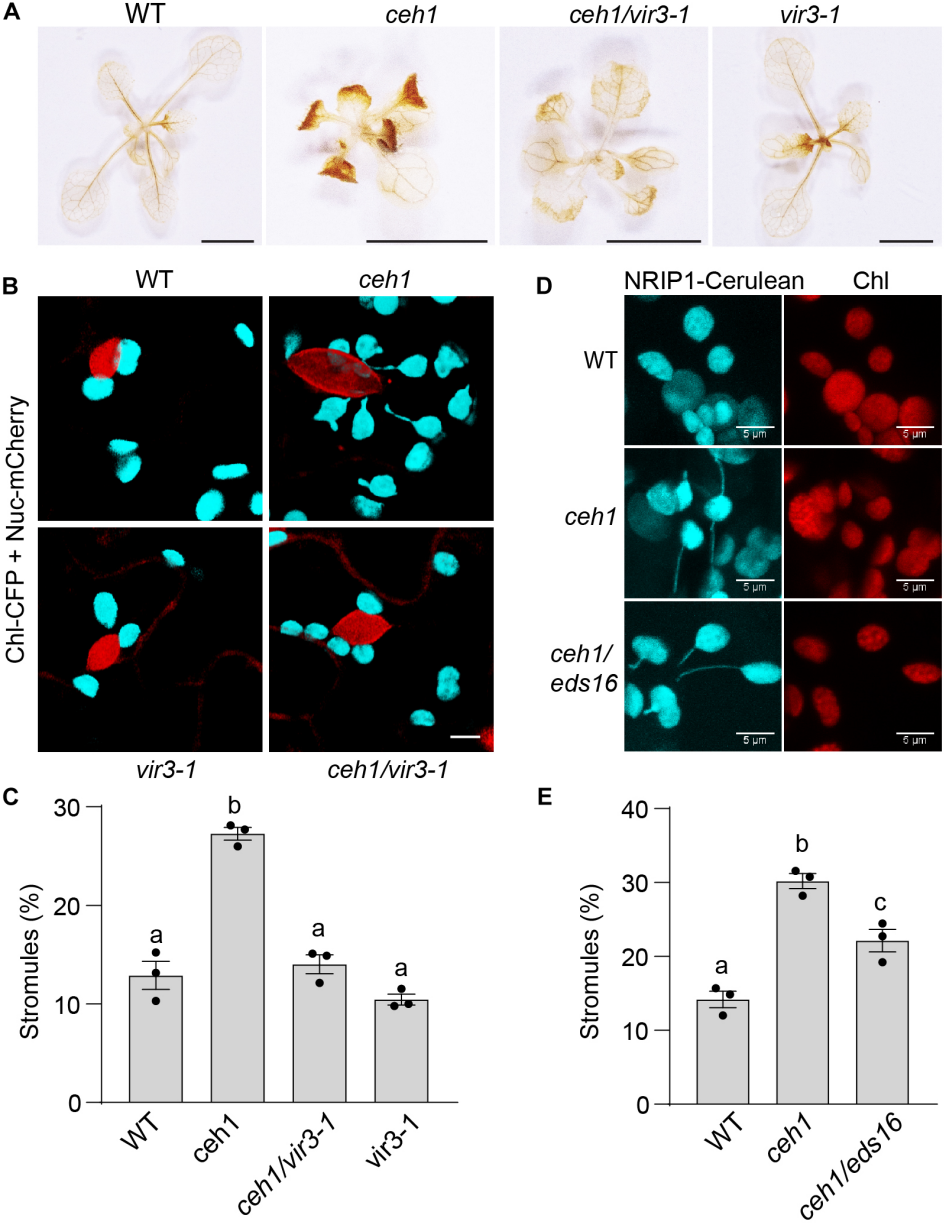
VIR3 functional input in ROS production and stromule formation. (**A**) Representative images of DAB-stained 2-week-old WT, *ceh1*, *ceh1/vir3-1*, and *vir3-1* seedlings show differential H_2_O_2_ accumulation in the genotypes. Scale bars, 0.5 cm. (**B** to **E**) Confocal images display nucleus (red) and chloroplast (cyan) without and with stromules in WT, *ceh1*, *ceh1/vir3-1*, and *vir3-1* (B), and (D) WT, *ceh1*, and *ceh1/eds16* seedlings with the images of chloroplast fluorescence, and their corresponding statistical analyses (C and E). The markers are CFP and mCherry fused to chloroplast transient peptide and to WPP nuclear localization signal, respectively. Scale bars, 5 μm. Data are means ± SEM (*n* ≥ 3) for each genotype repeated three times with similar results. Statistical analyses were performed by one-way ANOVA with Tukey’s multiple comparisons test (*P* < 0.05).

Given the tAPX function in intercepting H_2_O_2_ accumulation (Fig. 4I), we tested the consequential impact of reduced tAPX levels in *ceh1* compared to its seemingly stable levels in other genotypes (WT, *ceh1*/*vir3-1*, and *vir3-1*), using the 3,3′-diaminobenzidine (DAB) staining method (Fig. 5A and fig. S4A). The representative images showing the extent of DAB staining in the various seedlings are well aligned with differential abundance of tAPX in these lines, specifically confirming the higher H_2_O_2_ levels in *ceh1* compared to the other genotypes.

One of the reported functions of H_2_O_2_ is the induction of stroma-filled dynamic tubule structures that form in plastids, proposed to function as a conduit for transfer of information to nucleus (*35*, *36*). The differential levels of this signaling molecule prompted us to compare stromule formation in WT, *ceh1*, *ceh1*/*vir3-1*, and *vir3-1* genotypes transformed with a construct simultaneously expressing chloroplast-targeted cyan fluorescent protein (CFP) and nuclear-targeted mCherry (figs. S5, B and C, and S4B). The confocal images followed by statistical analyses portray a higher number of stromules formed in *ceh1* compared to the other three genotypes examined. Moreover, the statistically significantly higher number of stromules, in conjunction with increased SA levels in *ceh1*/*vir3-1* compared to WT and *vir3-1* genotypes (Figs. 1D and 5, B and C), led us to examine the potential contribution of SA to the formation of these conduits. Thus, we compared number of stromules formed in WT versus *ceh1* and the SA-deficient *ceh1* double mutant line (*ceh1/eds16-1*) lacking the SA biosynthesis gene (Fig. 5, D and E, and fig. S4, B and C) (*30*). The presented data are based on two independent lines each transformed with a different marker, namely, N receptor-interacting protein 1 (NRIP1)-Cerulean (*35*) and rubisco small subunit transient peptide (RB)-GFP (green fluorescent protein). Both lines show significantly fewer number of stromules in *ceh1/eds16* relative to the *ceh1*, but higher than the WT, establishing the previously reported contribution of SA to stromule formation (*35*).

The result collectively provides a direct link between the lowered tAPX enzyme abundance and the consequential higher H_2_O_2_ levels in conjunction with the increased number of stromules in the *ceh1* mutant. Furthermore, the finding establishes additive contributions of H_2_O_2_ and SA to the formation of these plastidial structures in the *ceh1* mutant backgrounds.

### HL recapitulates VIR3-mediated reconfiguration of the plastidial metabolic and structural states

**Fig. 6.**
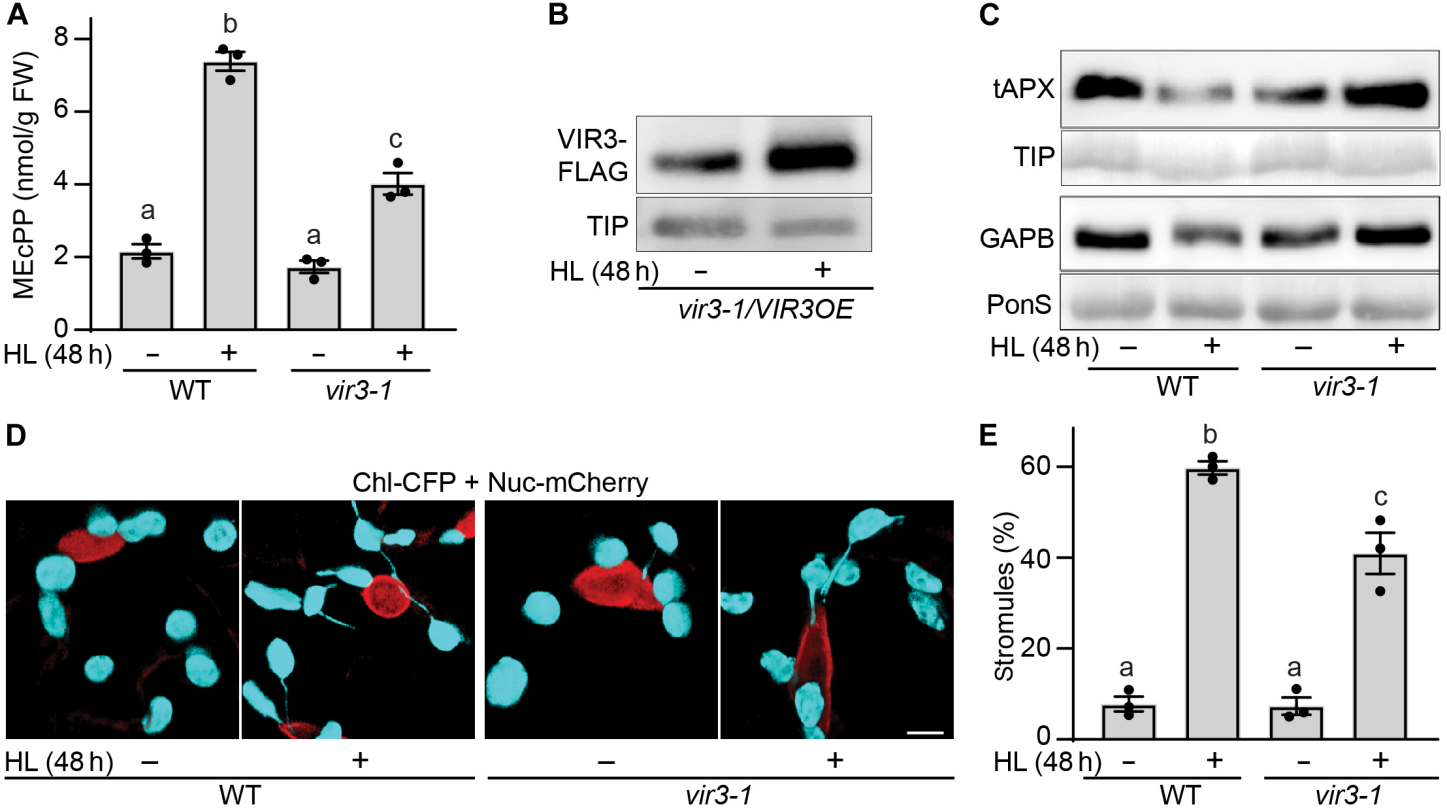
HL reaffirms VIR3 role in reconfiguration of the plastidial metabolic and structural states. (**A**) Light induction of MEcPP accumulation in untreated (−) and 48-hour HL-treated (+) WT and *vir3-1* seedlings. (**B**) Immunoblot analyses of VIR3 abundance using FLAG antibody in untreated (−) and 48-hour HL-treated (+) *vir3-1/VIR3OE* seedlings. TIP antibody was used on the same blot as the loading control. (**C**) Immunoblot analyses of tAPX and GAPB protein levels in untreated (−) and 48-hour HL-treated (+) WT and *vir3-1* seedlings, using TIP antibody and ponS as loading controls, respectively. (**D**) Representative confocal images showing nucleus (red) and chloroplasts (cyan) without and with stromules in untreated (−) and 48-hour HL-treated (+) WT and *vir3-1* seedlings and (**E**) their corresponding statistical analyses. Scale bar, 5 μm. Data are means ± SEM (*n* ≥ 3). The experiment was repeated three times for each genotype. Statistical analyses were performed by one-way ANOVA with Tukey’s multiple comparisons test (*P* < 0.05).

To examine VIR3 functional role in plant adaptive responses, we exploited HL as a naturally occurring prevalent daily challenge. Here, we show HL-mediated increase in MEcPP levels in WT and *vir3-1* mutant compared to the untreated lines (Fig. 6A). However, the notably higher MEcPP levels in the HL-treated WT versus that of *vir3-1* contradicts the earlier data (Figs. 1C and 2C), supported by the notion that the greater abundance of GAPB enzyme in *ceh1*/*vir3* mutant might increase the conversion of 1,3BPG to GAP and, by extension, enhance MEcPP levels (Fig. 1C). A possible reason for this discrepancy could be the transient accumulation of lower levels of MEcPP in HL-treated *vir3-1* compared to the constitutively higher levels of MEcPP in the *ceh1* mutant or to the high levels accumulated in the *HDSi* seedlings at 72 hours after DEX treatment. To examine this possibility, we measured MEcPP levels in *HDSi* and *HDSi/vir3-1* lines at 0 and 24 hours after DEX treatment. We reasoned that under these conditions, *HDSi* seedlings accumulate ~30-fold lower MEcPP levels and for a shorter period than those in the *ceh1* mutant and *HDSi* seedlings 72 hours after DEX treatment Figs. 1C and 2C and fig. S5). *HDSi/vir3-1* seedlings at 24 hours after DEX treatment accumulate lower MEcPP levels compared to those of the *HDSi*. The similarity of these data and those of HL-treated seedlings suggest a direct or an indirect VIR3 functional input in regulation of the MEP-pathway manifested by MEcPP levels.

Next, we examined the correlation between HL-mediated increase of MEcPP and the potential enhanced abundance of VIR3 as previously observed in MEcPP accumulating *ceh1* and *HDSi* lines (Fig. 4, B and C, and fig. S3, A and C). Because of the unavailability of a VIR3 specific antibody, and undetectable VIR3 levels in plants expressing VIR3-FLAG under the native promoter, HL assays were performed with *vir3-1*/*VIR3OE-FLAG* lines (Fig. 6B and fig. S6, A and B). The data show that even the overexpressing line exhibits higher levels of VIR3 in HL-treated versus untreated lines.

Next, we compared the abundance of tAPX and GAPB in WT and *vir3-1* plants subjected to no or 48 hours of HL treatment (Fig. 6C and fig. S6, C to E). The levels of both enzymes are reduced in the HL-treated WT seedlings but are increased in *vir3-1* mutant plant, supporting the inverse correlation between abundance of VIR3 and the levels of its interacting proteins.

Furthermore, confocal examination of stromule formation in untreated and HL-treated WT and *vir3-1* seedlings illustrate the presence of higher number of these dynamic structures in HL-treated seedlings, albeit more pronounced in the WT as compared with the *vir3-1* mutant (Fig. 6, D and E). These experiments reaffirm stress regulation of VIR3 abundance and the role of VIR3 in reducing the stability and/ or translational capabilities of GAPB and tAPX enzymes and the formation of plastidial structure, stromules.

## DISCUSSION

Dynamic regulation of plastids via complex and intertwined networks of sensing, metabolic, and signaling functions guides cellular homeostasis and shapes plant growth and development in an ever-changing environment. The data presented here reveal a multifaceted functional link between retrograde signaling and its signal transduction pathway component, VIR3. The finding specifically establishes stress-induced MEcPP-mediated increase in VIR3 levels and an inverse correlation between VIR3 abundance and the levels of its interacting proteins, GAPB and tAPX, and the resulting shifts in plastidial metabolic and structural states.

Specifically, ample evidence obtained from a suppressor screen and the results from various loss-of-function and gain-of-function genotypes, together with those of inducible MEcPP-producing lines (*HDSi* and *HDSi/vir3-1*), verifies VIR3 as a MEcPP signal transduction pathway component. Moreover, the significance of the zinc-binding domain in the VIR3 ability to complement the light-stress–induced variegation phenotype of *vir3-1* mutant corroborates the previous report using VIR3^H235L^ mutant construct (*10*). These data together with the identification of tAPX and GAPB as the VIR3 interacting proteins and the inverse correlation between their levels and the VIR3 abundance provide support for the metalloprotease nature of this enzyme. It is of note that the positive correlation between stress-induced accumulation of MEcPP and increased abundance of VIR3 is not supported by heightened transcript levels but by the enhanced translational capability and/or stability of VIR3 protein. This finding suggests a potential stress-induced MEcPP-mediated deactivation of a yet unknown protease that may target VIR3 under standard conditions.

Stress induction of MEcPP-mediated increase in VIR3 abundance results in reduced levels of tAPX, the enzyme responsible for detoxification of H_2_O_2_, a key signaling molecule with relatively long half-life that can alter cellular redox state and initiate Ca^2+^ signaling (*37*). Ca^2+^ signaling is necessary for induction of genes such as *ICS1*, whose expression is regulated by MEcPP-mediated induction of the cis-regulatory element *RSRE*, in a calcium-dependent manner (*27*). These reports support the previously reported dose-dependent increase of SA in response to increasing H_2_O_2_ concentration (*37*) and further offer a potential molecular mechanism for reduced SA levels in *Rceh4* and *ceh1*/*vir3-1* versus the heightened SA levels in *ceh1/vir3-1/VIR3OE* lines (Fig. 1D). Another consequence of increased H_2_O_2_ is inhibition of the HDS enzyme activity and the enhanced levels of MEcPP (*31*).

An added outcome of stress-induced MEcPP accumulation and heightened VIR3 levels is decreased levels of GAPB enzyme available for the conversion of 1,3BPG to GAP, resulting in an expected decreased level of GAP (Fig. 4I). This, in turn, could limit the MEP-pathway flux via reducing condensation of GAP with pyruvate, as the first step of the pathway (*23*–*26*). This decrease in flux may be a mechanism to constrain an excessive accumulation of MEcPP by the stress-induced reduced activity of the key enzyme (HDS) constitutively (*ceh1*) or for a long period (*HDSi* 72 hours after DEX treatment) (*29*–*31*, *34*, *38*–*41*). This delicate readjustment of substrate level in response to altered enzyme activity may provide an additional layer of complexity in regulating MEcPP production while maintaining the MEP-pathway flux for production of essential metabolites, isopentenyl diphosphates (IPPs). However, the inverse correlation between the GAPB abundance and levels of MEcPP accumulation in HL-treated WT versus *vir3-1* mutant plants (Fig. 6, A and C) support the involvement of additional regulatory steps, other than the fine-tuning of GAPB levels (Figs. 1C and 4F). One such mechanism could be kinetic inhibition/reduction of GAPB enzyme activity by ROSs that oxidize the catalytic cysteine in the enzyme active site. This notion is supported by the earlier reports of ROS inhibition of the cytosolic and plastidial GAPDH enzymes (*17*, *42*–*44*). In addition, stress-induced accumulation of ROS may, in turn, be regulated by GAPDHs, corroborating moonlighting function of these isozyme in response to biotic stresses (*22*). Collectively, the result alludes to multifaceted and complex regulatory mechanisms that differentially and delicately adjust MEcPP levels in plants, in accordance with constitutively (long-term stress) or transiently (short-term stress) reduced HDS enzyme activity.

The shift in H_2_O_2,_ MEcPP, and SA levels is accompanied by formation of stromules, plastidial structures formed in response to range of stresses, and postulated to function as a conduit for transfer of signaling factors from chloroplast to nuclei (*35*, *45*). The differential prominence of these structural phenotypes in *ceh1* versus *ceh1/eds16* (Fig. 5, D and E, and fig. S4, B and C), and between HL-treated WT and *vir3-1* mutant (Fig. 6, D and E), are likely due to accumulation of both SA and H_2_O_2_ as the result of MEcPP-mediated enhanced abundance and/or stability of VIR3 and the consequential reduced abundance of GAPB and tAPX enzymes.

**Fig. 7.**
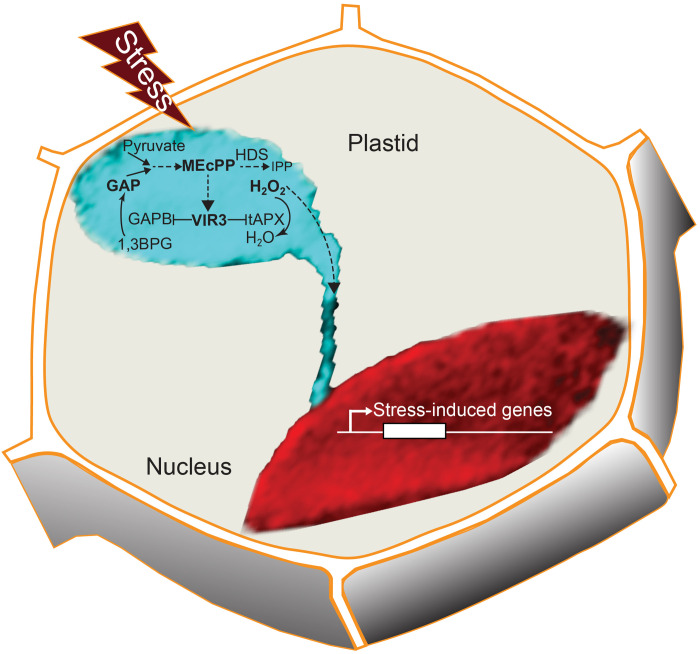
The simplified schematic model depicting the reciprocity between MEcPP and VIR3. The interlink between a plastidial stress-inducible retrograde-signaling metabolite, MEcPP, and a putative metalloprotease, VIR3, attunes the plastidial metabolic and structural states in response to environmental stimuli, enabling retrograde-mediated induction of selected nuclear-encoded stress-response genes.

The schematic model (Fig. 7) is a simplified depiction of the complex and intertwined reciprocity between MEcPP and VIR3 that provides a previously unrecognized link between the stress-induced plastidial retrograde signaling metabolite and a putative zinc-binding metalloprotease. This reciprocity provides insight into the molecular and biochemical venues involved in selective regulation of plastidial proteome and the consequential reconfiguration of the plastidial metabolic and structural states in response to environmental stimuli. This finding further signifies plastidial function in sensing multienvironmental inputs, integrating intraorganellar communication and ultimately mapping the cellular output central to maintenance of organismal integrity.

## MATERIALS AND METHODS

### Plant materials

All *Arabidopsis* lines used in this research are in the background of Columbia-0. The *ceh1* mutant and DEX-inducible MEcPP-accumulating line (*HDSi*) have been previously described (*30*, *46*). *Rceh4* mutant was obtained from the previously described screen by EMS mutagenesis (*46*). The *vir3-1* and *p35S::VIR3-FLAG/vir3-1* genotypes initially described (*10*) were provided by F. Yu (State Key Laboratory of Crop Stress Biology for Arid Areas and College of Life Sciences, Northwest Agriculture and Forestry University, People’s Republic of China). These lines were crossed with *pHPL::LUC*, *ceh1*, and *HDSi* and screened for homozygous lines. The *ceh1* and *ceh1*/*vir3-1* were also crossed with line expressing chloroplast CFP and mCherry-nuclear makers. Primers used for genotyping are listed in table S1.

Generation of *vir3-2* was based on CRISPR-Cas9 technology (*47*). Specifically, we designed single guide RNA (CCGTGGTGTGATATTGGATC) to target the fourth exon of *VIR3* (At1g56180) and cloned it into pEn-Chimera and recombined with pDe-Cas9 using Gateway cloning. The construct was introduced into *ceh1* via *Agrobacterium*-mediated transformation. The kanamycin-selected transformants were sequenced over the fourth exon to select CRISPR lines containing deletions or insertions. The next generation of transformants were selected for the mutation and Cas9 segregation.

The *VIR3^H239Y^*-FLAG construct was generated by site directed mutagenesis. Both *VIR3^H239Y^*-FLAG and *VIR3*-FLAG were amplified using *p35S::VIR3*-FLAG as a template and cloned into pENTR-D/Topo. All constructs were transferred to PUB-DEST vector to generate *pUBQ*::*VIR3^H239Y^*-FLAG and *pUBQ*::*VIR3*-FLAG constructs subsequently used to transform *vir3-1* line.

### Growth conditions

All seeds were surface-sterilized and sown on half-strength Murashige and Skoog medium [MS basal medium (2.16 g/liter), MES (1 g/liter, pH 5.7), and phytagar (8 g/liter)]. For selection, tissue culture medium was supplemented with kanamycin (50 mg/liter), glufosinate ammonium (10 mg/liter) (basta), or hygromycin (25 mg/liter). After stratification for 2 to 3 days at 4°C in the dark, plates were transferred to a growth chamber at 22°C with long day (16-hour light and 8-hour dark) and ~100 μmol m^−2^ s^−1^ light intensity. After 14 days, areal parts were harvested and immediately frozen in liquid nitrogen and stored at −80°C until analysis.

HL treatment was performed with plate-grown seedlings on half-strength MS at 22°C with 16-hour light/8-hour dark cycle and exposed to HL (800 to 900 μmol m^−2^ s^−1^ light intensity) for 2 days, followed by harvesting the areal parts after the treatment. Plants were grown in constant light at ~100 μmol m^−2^ s^−1^ light intensity. *N. benthamiana* (tobacco) seeds were sown on Sunshine mix1 and grown for 6 weeks at 23°C and in a long-day condition and at ~100 μmol m^−2^ s^−1^ light intensity.

### LUC split assay constructs

Upon amplification of *VIR3*, *VIR3^H239Y^*, tAPX (At1g77490), and GAPB (At1g42970), the fragments were cloned into pDONR207 and sequenced. The constructs were then transferred to vectors pMK7WG-cL-2, pMK7WG-nL-2, pMK7-cL-WG2, and pMK7-nL-WG2 using Gateway Technology, resulting to generation of constructs: *p35S*::*VIR3-cLUC*, *p35S*::*VIR3-nLUC*, *p35S*::*cLUC*-*VIR3*, *p35S*::*nLUC-VIR3*, *p35S*:: *VIR3^H239Y^-cLUC*, *p35S*:: *VIR3^H239Y^-nLUC*, *p35S*::*cLUC*- *VIR3^H239Y^*, *p35S*::*nLUC- VIR3^H239Y^*, *p35S*::*tAPX-cLUC*, *p35S*:: *tAPX-nLUC*, *p35S*::*cLUC*-*tAPX*, *p35S*::*nLUC-tAPX*, *p35S*::*GAPB-cLUC*, *p35S*:: *GAPB-nLUC*, *p35S*::*cLUC*-*GAPB*, *p35S*::*nLUC-GAPB*, *p35S*::*cLUC*, and *p35S*::*nLUC*.

### Marker lines generation, confocal microscopy, and data analyses

Nuclear markers were generated by amplification of the WPP (tryptophan-proline-proline) domain of RANGap1 from pGLP2::NTF and the mCherry from pBL14, flanked by the actin-2 (ACT2) promoter and 35*S* terminator amplified from pACT2::BirA. These four fragments and the pMDC123 vector linearized by Spe I and Asc I were assembled together by New England Biolabs (NEB) Gibson Assembly. Plastidial marker was generated by amplification of genomic DNA from pt-yk transgenic plants containing rubisco plastidial transit peptide fused to CFP cassette (pt-CFP) (*48*). Next, the two cassettes were assembled into pMDC123 vector by NEB Gibson Assembly to generate the nucleus-mCherry-pt-CFP marker construct, subsequently used for transforming into the *ceh1* background, and eventually transgressed into other genotypes. These genotypes were used in confocal microscopy imaging. In addition, we used RB-GFP and NRIP1-Cerulean–expressing lines, the gifts from S. Theg and S. Dinesh-Kumar at UC Davis.

An upright confocal microscope (Zeiss LSM 880 upright laser scanning confocal microscopy) equipped with 40×/1.2 water or 40×/1.4 oil objectives was used for fluorescent protein marker detection. Confocal images were collected through *z*-stacks on Zeiss LSM 880, and the number of stromules was counted per field of view as described (*49*, *50*). More than three seedlings were used for these analyses.

### MEcPP and SA measurements

Extraction and quantifications of MEcPP and SA were performed as previously described (*33*, *46*).

### Luciferase (LUC) measurements

Fourteen-day-old plants grown on half-strength MS were sprayed with 1 mM Luciferin (Gold Biotechnology, LUCK-1G) with 0.001% Triton X-100 and immediately imaged 10× for 15 min each time using an Andor DU434-BV charge-coupled device camera (CCD) (Andor Technology). LUC activity was quantified as described before (*51*, *52*).

### Tobacco infiltration

Tobacco leaves of 6-week-old plants were infiltrated as described previously (*51*). Cultures of constructs grown in *Agrobacterium tumefaciens* GV3101 were prepared in infiltration medium [50 mM MES (pH 5.6), 9.8 mM MgCl_2_-6H_2_O, 27.7 mM d-glucose, and 0.1 mM acetosyringone]. Before infiltration, cultures were diluted to an optical density at 600 nm of 0.1 and mixed in equal amounts. P19 was infiltrated with all samples.

### IP assay

IP was performed according to the reported method (*53*), an amenable method for isolation of membrane proteins, using tobacco leaves 72 hours after infiltration, which were harvested and immediately frozen in liquid nitrogen. Proteins were extracted in extraction buffer [25 mM tris-HCl (pH 7.5), 150 mM NaCl, 10% glycerol, 0.1% Triton X-100, 2% polyvinylpolypyrrolidone, 10 mM dithiothreitol, and 1% proteinase inhibitor without EDTA] and centrifuged for 10 min at 14,000 rpm, and the supernatant was incubated with 10 μl of packed anti-FLAG M2 beads (MilliporeSigma) for 4 hours, at 4°C. Beads were washed 4× with wash buffer [10% glycerol, 25 mM tris (pH 7.5), 150 mM NaCl, 2% polyvinylpolypyrrolidone, and 10 mM dithiothreitol] and 1× with 1 ml of ammonium bicarbonate solution (50 mM, pH 8). On-beads trypsin digestion was performed as previously described (*54*).

IP assays using *Arabidopsis* lines were performed on proteins obtained from enriched chloroplasts as previously described (*55*). In short, areal tissue of 14-day-old plants grown on half-strength MS was homogenized in extraction buffer [1.25 M NaCl, 0.25 M ascorbic acid, 10 mM sodium metabisulfite, 0.0125 M sodium tetraborate, 50 mM tris (pH 8.0), 1% PVP-40 (polyvinylpyrrolidone, average 40,000 molecular weight), 0.1% bovine serum albumin (BSA), and 1 mM dithiothreitol (pH 3.8)]. The homogenate was filtered through Miracloth and centrifuged at 200*g* for 20 min, followed by centrifugation of supernatant at 200*g* for 20 min and thereafter at 3500*g* for 20 min. The pellet was washed with wash buffer [1.25 M NaCl, 0.0125 M sodium tetraborate, 50 mM tris (pH 8.0), 1% PVP-40, 0.1% BSA, and 1 mM dithiothreitol (pH 8.0)] and centrifuged at 3750*g* for 20 min. The pellet was resuspended in IP buffer (1× PBS, 0.3% Triton X-100, and 1% proteinase inhibitor without EDTA) and mixed with anti-FLAG M2 beads and continued as described above. After the fourth wash, beads were mixed with 2× SDS loading buffer and used in immunoblot analyses.

### Protein extraction and immunoblot analysis

Soluble and membrane-bound proteins were extracted from 14-day-old areal tissue according to the previously described method (*56*). Soluble proteins were separated on a 12% SDS–polyacrylamide gel electrophoresis (SDS-PAGE) gel, and membrane proteins were loaded on a 15% urea–SDS-PAGE gel as described (*57*), and subsequently transferred onto polyvinylidene difluoride (PVDF) membranes. Membranes with soluble proteins were probed with anti-GAPB (a gift from S. Zeeman, ETH Zurich, Switzerland) (1:3000), whereas membrane protein blots were probed with anti-APX (Agrisera, AS08 368) (1:2000) and with anti-FLAG M2 (MilliporeSigma) (1:1000) or anti-αTIP (alpha tonoplast intrinsic protein, AT1g73190) (Arabidopsis Biological Resource Center, AB00111) (1:1000). Accordingly, the selected secondary antibodies included anti-rabbit horseradish peroxidase (HRP) (Thermo Fisher Scientific, 31460) (1:20,000), anti-mouse HRP (KPL, 074-1806) (1:10,000), anti-chicken HRP (KPL, 14-24-06) (1:10,000), or anti-chicken phosphatase (KPL, 15-24-06) (1:3000). Visualization was by chemiluminescent reaction substrate (Thermo Fisher Scientific, SuperSignal West Pico Plus chemiluminescent substrate) according to the manufacturer’s instructions and ChemiDoc PM (Bio-Rad). Protein band intensities were quantified and normalized using Image Lab 6.0.1 software (Bio-Rad).

### Proteomics

A two-dimensional nano LC-MS analysis of trypsin-digested proteins was performed as previously described (*54*, *58*). Proteome Discoverer 2.1 (Thermo Fisher Scientific) was used to analyze the raw MS data files. MS data were matched to Niben1.0.1 and NbS tobacco, TAIR10 *Arabidopsis*, and SL4.0 tomato databases (data S1).

### Quantitative gene expression analyses

Total RNA was extracted and used for real-time quantitative reverse transcription polymerase chain reaction (real-time qRT-PCR) as previously described (*34*). The normalization was carried out using the internal control AT4G26410. Each experiment was performed on three biological replicates each with three technical replicates. Primers used to detect VIR3, tAPX, and GAPB were listed in table S1.

### Statistical analyses

All experiments were performed with at least three biological replicates. Data are means ± SD. The analyses were carried out via a two-tailed Student’s *t* test with a significance of *P* < 0.05.

The primers used in the construction are summarized in table S1.

## Notes

View/request a protocol for this paper from *Bio-protocol*.
