# Supplementary material for: Reciprocity between a retrograde signal and a putative metalloprotease reconfigures plastidial metabolic and structural states
Source: Sci Adv. 2022 Jun 3;8(22):eabo0724. doi: 10.1126/sciadv.abo0724 (PMC9166295; doi:10.1126/sciadv.abo0724)
Supplement: Supplementary file 1 — Figs. S1 to S6 Table S1 [file sciadv.abo0724_sm.pdf]

Science Advances
AAAS

# Supplementary Materials for

## Reciprocity between a retrograde signal and a putative metalloprotease reconfigures plastidial metabolic and structural states

Jin-Zheng Wang *et al.*

Corresponding author: Katayoon Dehesh, kdehesh@ucr.edu

*Sci. Adv.* **8**, eabo0724 (2022)
DOI: 10.1126/sciadv.abo0724

**The PDF file includes:**

Figs. S1 to S6
Table S1
Legend for data S1

**Other Supplementary Material for this manuscript includes the following:**

Data S1

**a**

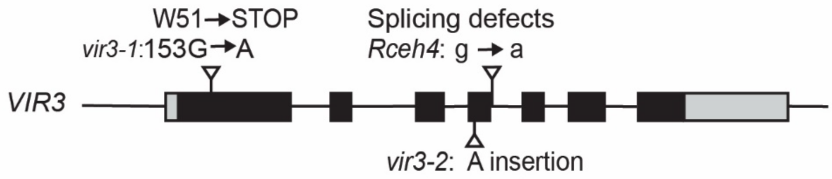


**b**

*Rceh4*: g → a

WT: CCGTGGTGTGATATTGGATCCGGTTGTTGCCATGCAAATGGGGGTTCAAGGCCAG gtattattt

**gRNA**

*vir3-2*: CCGTGGTGTGATATTGG**A**ATCCGGTTGTTGCCATGCAAATGGGGGTTCAAGGCCAG gtattattt

**c**

*Rceh4*: g → a

WT-DNA: GTTCAAGGCCAG **g**tattattt...tctctag GCTGGA

WT-mRNA: GTTCAAGGCCAG GCTGGA

Amino acids: V Q G Q A G

*Rceh4*-mRNA: GTTCAAGGCCAG atattattt...tctc**tag** GCTGGA

Amino acids: V Q G Q I L .............*

**d**

WT-DNA: CCGTGGTGTGATATTGGATCCGGTTGTTGC

WT-mRNA: CCGTGGTGTGATATTGGATCCGGTTGTTGC

Amino Acids: R G V I L D P V V A

*vir3-2*-mRNA: CCGTGGTGTGATATTGG**A**ATCCGGTTGTTGC

Amino Acids: R G V I L **E S G C C**

**Supplemental Fig. S1. *VIR3* mutant alleles**

(**a**) Schematics of *VIR3* gene and the display of the location of various mutant alleles with annotation of the corresponding changes in the nucleotides and their coded amino acids. (**b**) Sequence of guard RNA (gRNA) resulting in the insertion of an additional nucleic acid (A) in VIR3 DNA sequence, used for generating *vir3-2* mutant line. (**c**-**d**), Nucleotide sequences and the corresponding amino acids of WT and mutant alleles of VIR3.

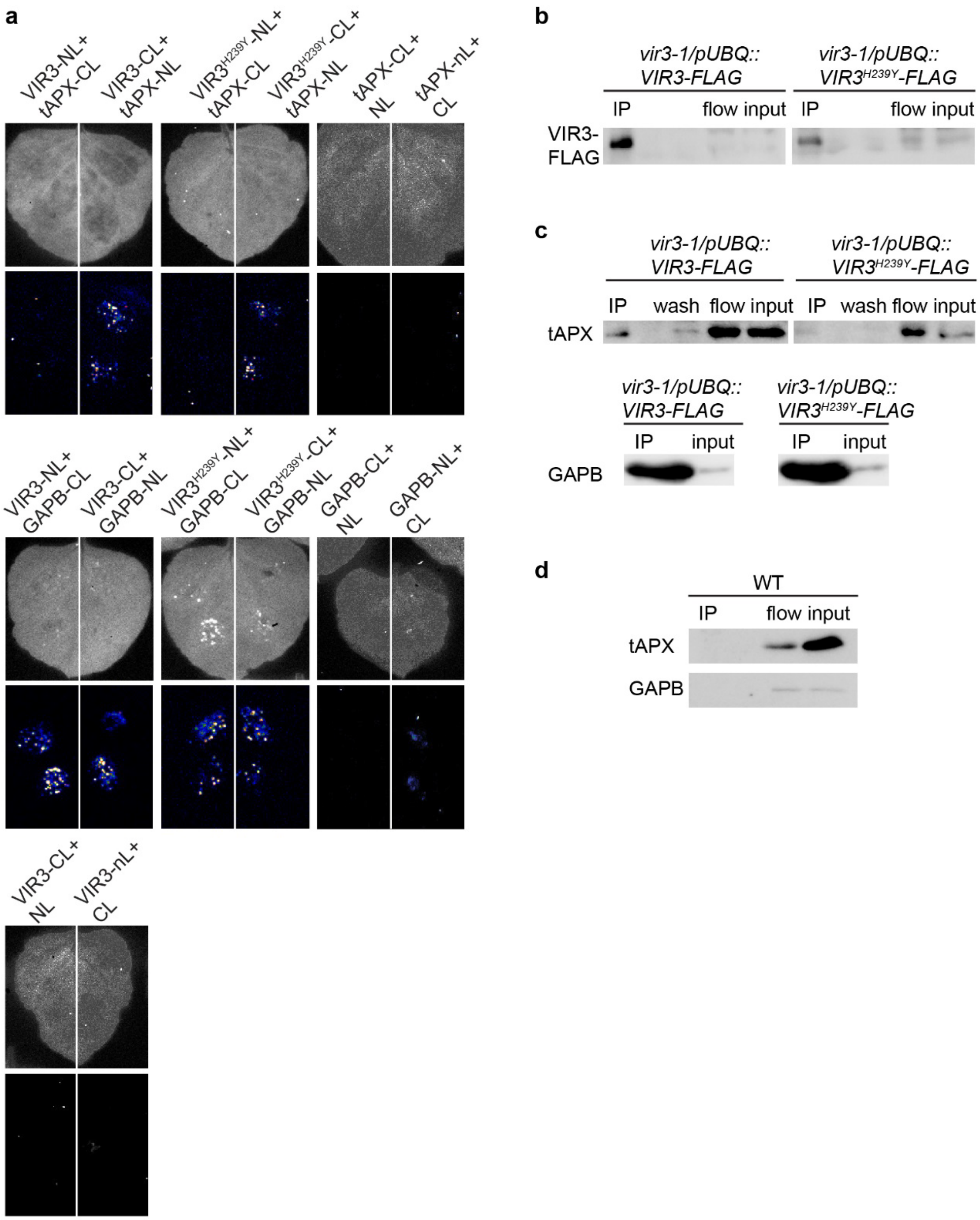


**Supplemental Fig. S2. Integrity of zinc-binding motif is not essential for VIR3 binding to tAPX and GAPB.**

(**a**) Representative images of light-field (top panels) and dark-field (bottom) showing split luciferase complementation assays in N. benthamiana leaves expressing permutations of VIR3 or VIR3$^{H239Y}$ with tAPX (upper panels) or GAPB (lower panels), each fused to N- and C-terminal fragments of luciferase (NL and CL respectively). Control consisted of fusion constructs between tAPX and GAPB with luciferase only. The experiments were repeated at least three times. (**b**) Immunoprecipitation (IP) assay using FLAG antibody displays presence of VIR3 in both *vir3-1/pUBQ::VIR3-FLAG* and *vir3-1/pUBQ::VIR3$^{H239T}$-FLAG* transgenic line. (c) Co-immunoprecipitation (Co-IP) show the in vivo physical interaction between VIR3 with tAPX and GAPB in *vir3-1/-pUBQ::VIR3-FLAG* and *vir3-1/pUBQ::VIR3$^{H239T}$-FLAG* seedlings, using FLAG antibody for Co-IP, tAPX and GAPB specific antibodies used on the immunoblots. (d) Immunoblot analyses using tAPX and GAPB specific antibodies on beads incubated with proteins extracted from the WT seedlings.

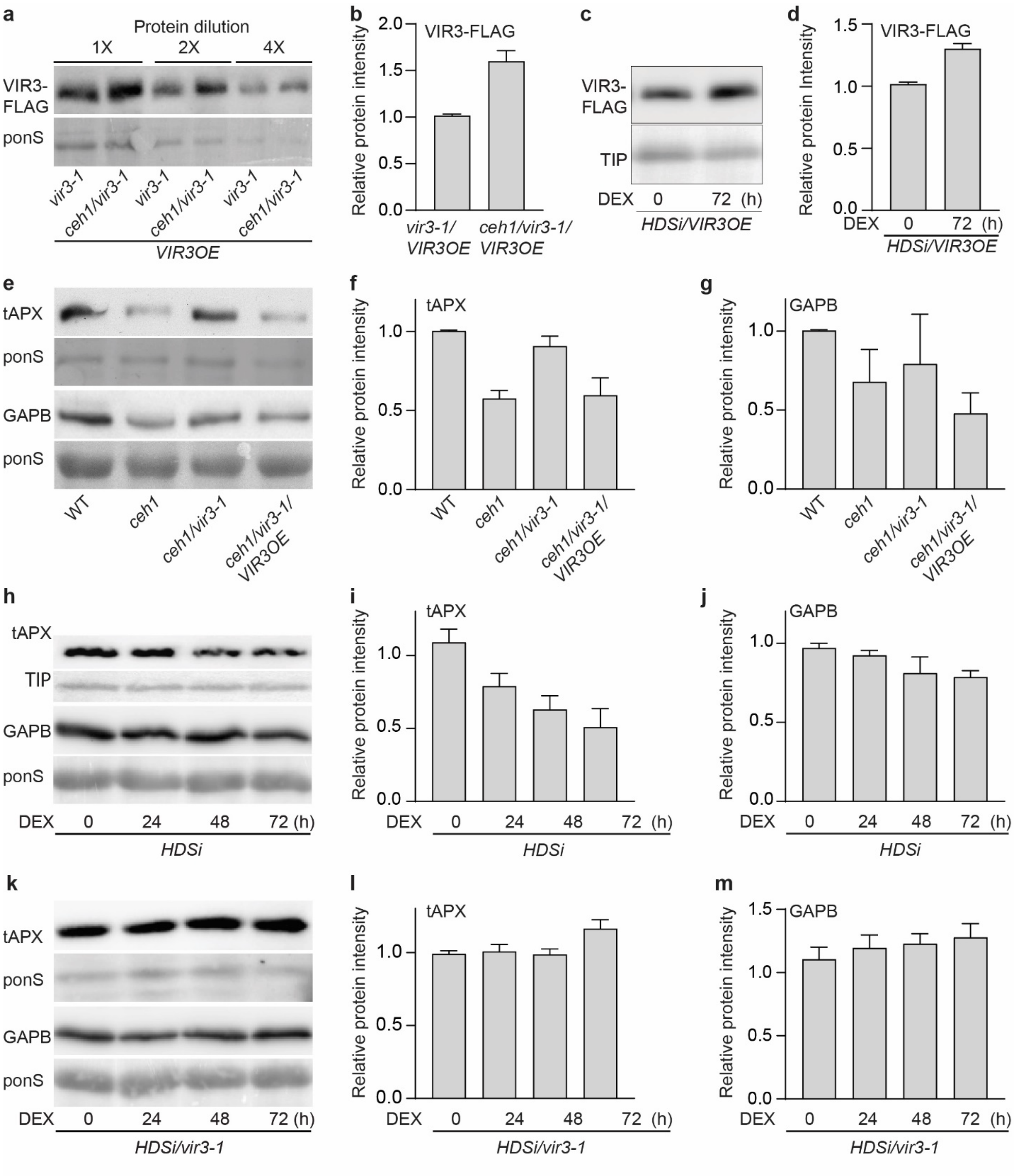


**Supplemental Fig. S3. MEcPP-mediated increase in VIR3 is inversely correlated with tAPX and GAPB abundance**
(**a**-**b**) Immunoblot analyses of protein dilution series probed with FLAG antibody (top) and ponceau staining (ponS) as the loading control (bottom) to show abundance of VIR3-FLAG in *vir3-1* and *ceh1/vir3-1* seedlings (**a**) and the corresponding intensity measurements using BioRad 6.0.1 software program (**b**). (**c**-**d**) Immunoblot analyses of VIR3 abuandce in untreated (0) and DEX-treated (72h) *HDSi/VIR3OE* seedlings proteins using FLAG antibody (top) and TIP antibody as the loading control (bottom) (**c**), and the corresponding intensity measurements using BioRad 6.0.1 software program (**d**). (**e**-**m**) Immunoblot analyses of tAPX (top panels), and GAPB (lower panels) in WT, *ceh1*, *ceh1/vir3-1* and *ceh1/vir3-1/VIR3OE* (**e**), and in *HDSi* (**h**) and *HDSi/vir3-1* genotypes (**k**), at various hours post DEX-treatment (0, 24, 48 and 72 hours), using TIP and ponS as loading controls respectively. The respective protein intensity measurements using BioRad 6.0.1 software program (**f**-**g**, **i**-**j** and **l**-**m**) are shown.

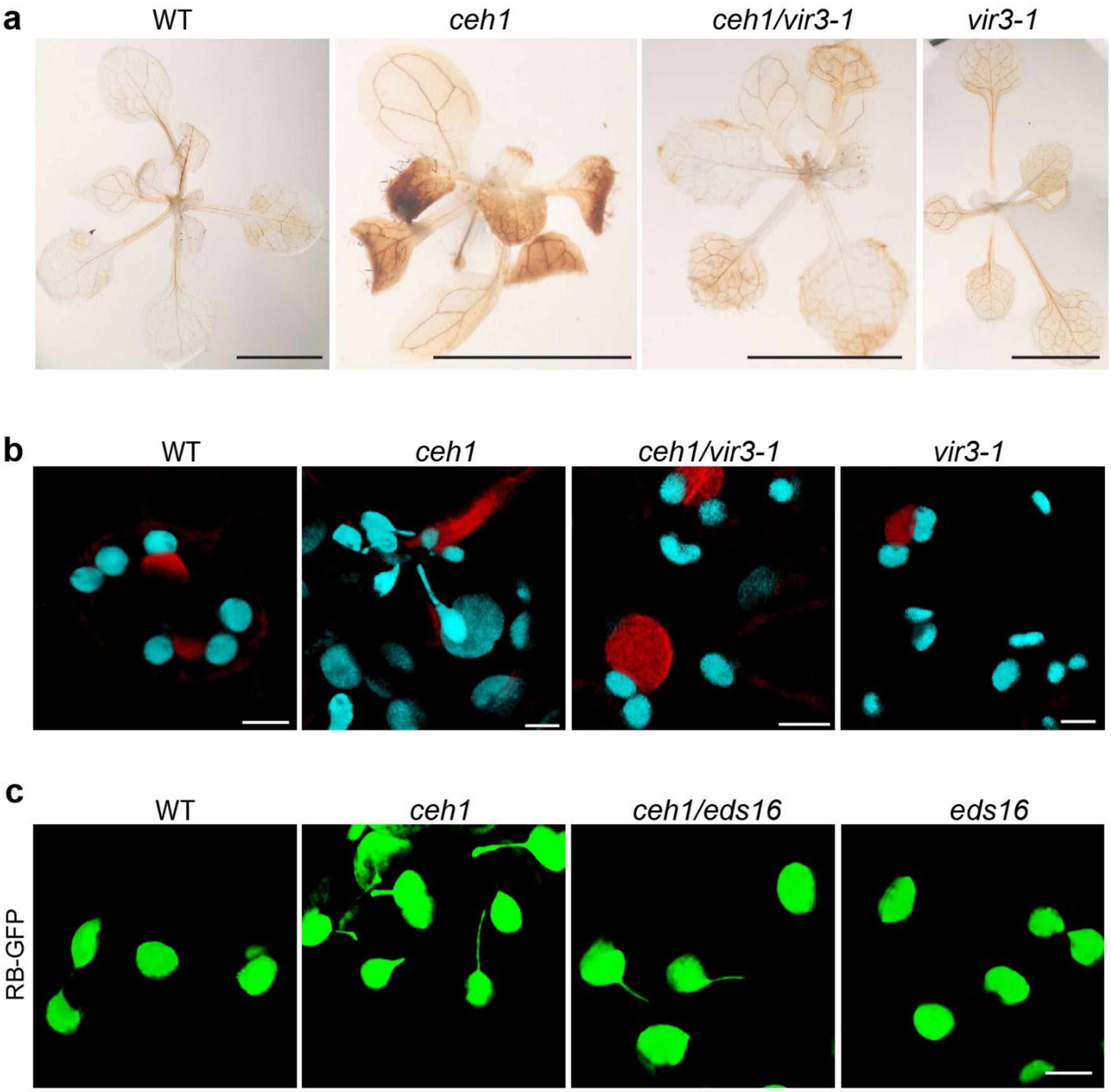


**Supplemental Fig. S4. VIR3 functional input in ROS production and stromules formation**

(**a**) Representative images of diaminobenzidine (DAB) stained 2-week-old WT, *ceh1*, *ceh1/vir3-1* and *vir3-1* seedlings show differential $H_2O_2$ accumulation in the genotypes. Bar=0.5cm. (**b**) Confocal images display nucleus (red) and chloroplast (cyan) without and with stromules in transgenic WT, *ceh1*, *ceh1/vir3-1* and *vir3-1* expressing CFP and mCherry fused to chloroplast transient peptide and to WPP nuclear localization signal respectively. (**c**) Confocal images display chloroplasts (green) without and with stromules and in transgenic WT, *ceh1* and *ceh1/eds16* seedlings expressing RB-GFP. Bar=5µm.

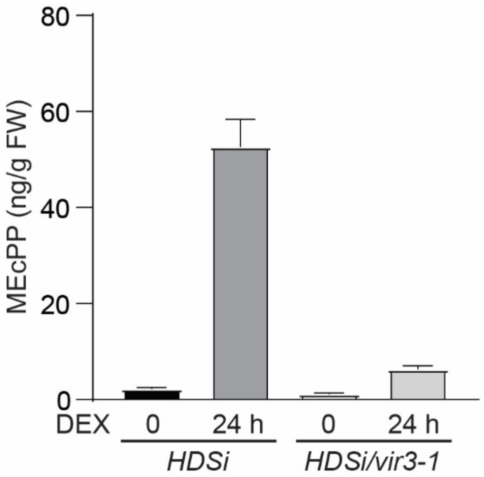


**Supplemental Fig. S5. VIR3 and MEcPP production.**
(**a**) MEcPP levels in 2-week-old untreated (0) or DEX-treated (post 24 h) *HDSi* and *HDSi/vir3-1* seedlings grown under long day conditions. Data are mean ± SD for each genotype with at least three biological replicates.

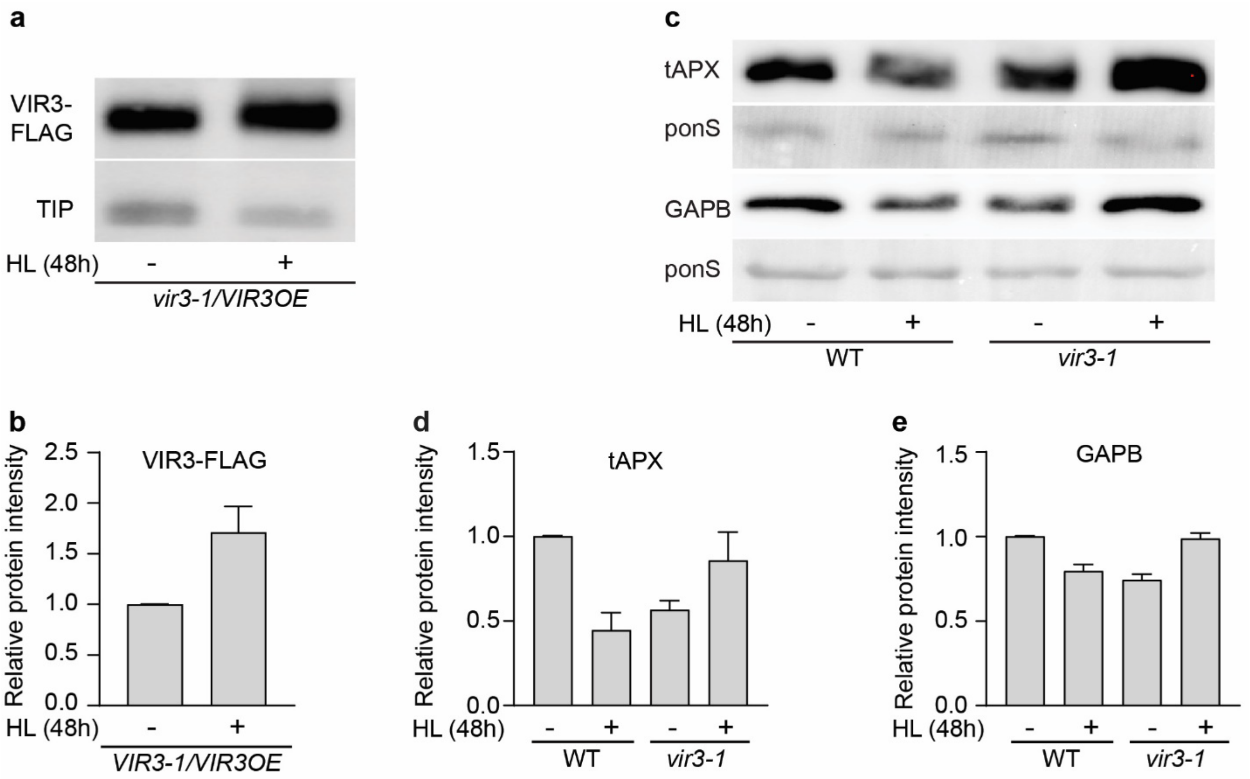


**Supplemental Fig. S6. High-light induction of VIR3 protein abundance and reduction of tAPX and GAPB protein levels**

(**a**-**b**) Immunoblot analyses of VIR3 abundance using FLAG antibody in untreated (-) and 48 h high-light (HL)-treated (+) *vir3-1/VIR3OE* seedlings (**a**), and the corresponding relative intensities using BioRad 6.0.1 software program analyses (**b**). TIP antibody was used on the same blot as the loading control. (**c**-**e**) Immunoblot analyses of tAPX (upper panel) and GAPB (lower panels) protein levels in untreated (-) and 48h HL-treated (+) WT and *vir3-1* seedlings, using ponceau staining (ponS) as loading controls respectively, and the corresponding relative intensities (**d**-**e**).

Supplemental Table S1. List of Primers

| Primers used | | |
|---|---|---|
| primer name | primer sequence | notes |
| ceh1-F2 | 5'-TAT GTA GGT GGT TCT CCC GGA AAA ATC GAT-3' | dCAPs marker with MboI |
| ceh1-R1 | 5'-CCA CCC ATC TTT CTC CGT TTT GAA ATC TAC-3' | |
| VIR3-F1 | 5'-GCT TTA CTC GGC CCA ATG A-3' | dCAPs marker with FauI |
| VIR3-R1 | 5'-CAG TTC AAA TGC TCG CAC AC-3' | |
| VIR3-Flag-F | 5'-TAG GCT GGA ACC CAA TTC TG-3' | |
| VIR3-Flag-R | 5'-CTA CTT ATC ATC ATC TTT ATA ATC TTT-3' | |
| VIR3-crispr-F | 5'-ATT GCCG TGG TGT GAT ATT GGA TC-3' | |
| VIR3-crispr-R | 5'-AAA CGA TCC AAT ATC  ACA CCA CGG-3' | |
| VIR3-H239Y-F | 5'-CTG GTT ATC TTC TTG TTG CAT ACC T-3' | Site-directed mutagenesis |
| VIR3-H239Y-R | 5'-GGT ATG CAA CAA GAA GAT AAC CAG-3' | |
| VIR3-F | 5'-CAC CAT GGC TTT ATC TCC GTC GTC-3' | |
| VIR3cDNA-attB1 | 5'-GGG GAC AAG TTT GTA CAA AAA AGC AGG CTT AAT GGC TTT ATC TCC GTC GTC-3' | |
| VIR3cDNA-attB2 | 5'-GGG GAC CAC TTT GTA CAA GAA AGC TGG GTT TTT GCT TGA AGA CAT GGC TTC CTC-3' | |
| tAPXcDNA-attB1 | 5'-GGG GAC AAG TTT GTA CAA AAA AGC AGG CTT AAT GTC TGT TTT CTC TCC GC-3' | |
| tAPXcDNA-attB2 | 5'-GGG GAC CAC TTT GTA CAA GAA AGC TGG GTT TTA GAA ACC AGA GAA ATC GGA GTT G-3' | |
| GAPBcDNA-attB1 | 5'-GGG GAC AAG TTT GTA CAA AAA AGC AGG CTT AAT GGC CAC ACA TGC AG-3' | |
| GAPBcDNA-attB2 | 5'-GGG GAC CAC TTT GTA CAA GAA AGC TGG GTA GTC ATA GAC TTT GCA TTC CTC ATC-3' | |
| VIR3-qrt-F | 5'-CAT GCT ACT GGC CTC CAC AT-3' | |
| VIR3-qrt-R | 5'-TAC CGC TAA GTC GTC CCT CA-3' | |
| tAPX-qrt-F | 5'-GCT AGT GCC ACA GCA ATA GAG GAG-3' | |
| tAPX-qrt-R | 5'-TGA TCA GCT GGT GAA GGA GGT C-3' | |
| GAPB-qrt-F | 5'-CGG TGG GGA AGC ATC TTT CT-3' | |
| GAPB-qrt-R | 5'-CGC AAC TTT CAG TTT CGC CA-3' | |
| GF1 | GGCGGCCGCTCTAGAACGACAAAATTTAGAACGAAC | Nuclues-mCherry marker |

| | | |
|---|---|---|
| GR1 | CCATTTTTTATGAGCTGCAAACACAC | Nuclues-mCherry marker |
| GF2 | GCAGCTCATAAAAAATGGATCATTCAGCGAAAACC | Nuclues-mCherry marker |
| GR2 | ACCATTGCTGCAGCCTCAACCTCGGATTCTTC | Nuclues-mCherry marker |
| GF3 | TGAGGCTGCAGCAATGGTGAGCAAGGGCGA | Nuclues-mCherry marker |
| GR3 | TGGAGATTACTTGTACAGCTCGTCCATG | Nuclues-mCherry marker |
| GF4 | CTGTACAAGTAATCTCCATAATAATGTGTGAG | Nuclues-mCherry marker |
| GR4 | GGGCCCCCCCTCGAGGCGACGGGGATCTGGATTTTA | Nuclues-mCherry marker |
| GF5 | GGCGGCCGCTCTAGAACCGATCTAGTAACATAGATGAC | nucleus-mCherry-pt-CFP marker |
| GR5 | GTCGTACTCCAAAAATGTCAAAGATACAGTCTC | nucleus-mCherry-pt-CFP marker |
| GF6 | ACATTTTTGGAGTACGACAAAATTTAGAACGAAC | nucleus-mCherry-pt-CFP marker |
| GR6 | TGGAGTACGACGGGGATCTGGATTTTAGT | nucleus-mCherry-pt-CFP marker |
| GF7 | TCCCCGTCGTACTCCAAAAATGTCAAAGATACAGTC | nucleus-mCherry-pt-CFP marker |
| GR7 | GGGCCCCCCCTCGAGGCCGATCTAGTAACATAGATGAC | nucleus-mCherry-pt-CFP marker |

**Supplemental Data S1. VIR3 interacting proteins**

Top candidates of VIR3 interacting proteins identified by Liquid Chromatography Mass Spectrometry-based proteomics
